# Low BMI and low TSH value as risk factors related to lower bone mineral density in postmenospausal women under levothyroxine therapy for differentiated thyroid carcinoma

**DOI:** 10.1186/s13044-015-0019-1

**Published:** 2015-06-02

**Authors:** Thaís Gomes de Melo, Lígia Vera Montalli da Assumpção, Allan de Oliveira Santos, Denise Engelbrecht Zantut-Wittmann

**Affiliations:** Division of Endocrinology, Internal Medicine Department, University of Campinas, Campinas, Brazil; Division of Nuclear Medicine, Radiology Department, University of Campinas, Campinas, Brazil

**Keywords:** Bone mineral density, Thyroid cancer, Thyrostimulating hormone, Bone mass index

## Abstract

**Objective:**

Treatment of differentiated thyroid carcinoma (DTC) includes suppression of TSH with levothyroxine therapy, which may negatively influence bone mineral density (BMD), but the effects are controversial. We aimed to evaluate the relationship between TSH-suppressive therapy and BMD in postmenopausal women with DTC.

**Methodology:**

Cross-sectional study that assessed BMD by densitometry and risk factors for decreased BMD in 109 postmenopausal women under TSH-suppressive therapy for DTC, compared to an age-matched euthyroid women control group. Conditions that might have affected BMD were exclusion criteria.

**Results:**

Patients were 58.4 ± 8.3 years-old, mean serum TSH was 0.21 ± 0.28μIU/ml. In BMD evaluation, T-scores were −1.09 ± 1.43 SD (lumbar spine) and −0.12 ± 1.18 SD (total femur). No significant differences were found between lumbar or femoral T-scores of patients and control group. Multivariate logistic regression analysis evidenced that low BMI and low mean TSH levels (assessed in the year of BMD measurement) were factors significantly related to lower lumbar and spinal BMD.

**Conclusion:**

Although low TSH levels and low BMI were correlated with lower BMD, it was not observed an increased prevalence of osteopenia or osteoporosis in this cohort of post-menopausal women under levothyroxine treatment for DTC, when compared to age-matched control women. Nevertheless, such risk factors should be carefully observed in individual patients at high risk of decrease in BMD.

## Background

Suppression of thyroid-stimulating hormone (TSH) with supraphysiological doses of levothyroxine (LT4) is one component of the treatment of differentiated thyroid carcinoma (DTC), after surgery and radioiodine therapy, aiming to reduce the risk of tumor recurrence [1]. In recent years, guidelines for TSH suppression therapy in the treatment of DTC have changed, due to the excellent prognosis of the tumor and better understanding of its course [2]. TSH suppression is recommended, but there is still no consensus about the ideal concentration of TSH for DTC patients. Besides the beneficial effects of TSH suppression in reducing tumor recurrence, potential risks should be considered [2].

The deleterious consequences of TSH suppression in the cardiovascular system are well-established [2, 3], but subclinical thyrotoxicosis may also have a negative influence on bone metabolism. Nevertheless, the magnitude of the effect and influence of additional factors on bone mineral density (BMD) of those patients remain unclear. Published data about the implications of TSH suppression on BMD in patients with DTC are conflicting [2]. Particularly in postmenopausal women, several authors have reported a negative effect of long-term TSH suppressive treatment on the BMD of patients with DTC [4–7], while other studies have not confirmed such negative effect [8–11]. BMD analysis is important because it is correlated with the risk of fractures in postmenopausal women [12]. We have not found studies with definite results about the correlation of TSH suppressive therapy for DTC and osteoporosis or osteopenia in post-menopausal women [13].

## Methods

The objective of this study was to evaluate the relationship between TSH suppressive therapy and BMD in postmenopausal women with DTC.

This investigation was a cross-sectional study with 109 postmenopausal women from a single center specialized in treatment of thyroid neoplasia. Patients were in follow-up for DTC, according to updated guidelines during the years. It is important to mention that the guidelines for treating DTC have changed along the time and some recommendations, such as the target TSH level, were modified during patients follow-up. All patients had their BMD evaluated between 2009 and 2011, in one single time. Patients were compared to a control group composed of postmenopausal euthyroid women, matched by age at the time of BMD assessment. The control group was obtained from the database of the bone densitometry unit of the Division of Nuclear Medicine in the same institution. Women with hyperthyroidism or hyperprolactinemia, permanent post-surgical hypoparathyroidism, intestinal malabsorption, rheumatoid arthritis, osteoarthritis, chronic kidney disease, prolonged immobilization, other malignant neoplasia or chronic use of corticosteroid and anticonvulsivants anytime during their lifetime were excluded from both groups, due to potential interference in BMD. After signing the informed consent form, patients were surveyed in order to assess risk factors that could be associated to low BMD, according to the National Osteoporosis Foundation [12] and the World Health Organization (WHO) guidelines [14]. Questions included smoking history, family history of osteoporosis, age at menarche and menopause, previous use of oral contraceptives, corticosteroids and anticonvulsants drugs, hormone replacement therapy for menopause, physical activity, and daily calcium intake. The study was approved by the local Ethics Committee, according to the 3^rd^ edition of the Guidelines on the Practice of Ethical Committees in Medical Research.

Lumbar spine and femoral BMD were measured with a “Hologic DXA Discovery Wi” densitometer. We analyzed lumbar spine (L1-L4) and total femur T-scores, and diagnosis was done according to the WHO criteria for the diagnosis of osteoporosis and osteopenia in postmenopausal women [12, 14].

We also analyzed TSH levels (chemiluminescence method, *sandwich* technique, Elecsys TSH-Roche, reference value: 0.40–4.5 μIU/ml) and free thyroxine (FT4-electrochemiluminescence method, competition principle, Elecsys FT4-Roche, reference value: 0.9–1.8 ng/dl). All TSH and FT4 levels values were obtained in the year previously to the BMD assessment for each patient were included in the analysis (ranging from 3 to 4 values for each patient). Besides that, we considered for the study all TSH level values in each patient’s file in order to evaluate the period of time each patient was exposed to different degrees of TSH suppression (<0.001 μIU/ml or <0.1 μIU/ml). Serum PTH was assessed in 80 patients by electrochemiluminescence method, reference value of 15-65 pg/ml.

Sample size calculation was done after a pilot analysis of 20 patients and 20 control individuals. Based on that, the minimal number of subjects for each group was 90 (alfa and beta error of 0.05 and 0.2, respectively). For the comparison of categorical variables between the main groups, we used Pearson’s chi-square test. Mann–Whitney test was used for the comparison of numerical variables between the two groups. The Spearman correlation coefficient was used to analyze the relationships between numerical variables. For the study of factors related to the BMD values, linear regression analysis was used, with univariate and multivariate models and stepwise criteria for variable selection. The statistical tests were performed in the SAS 9.1.3 System (SAS Institute Inc, 2002–2003, Cary, NC, USA), and the level of significance for the statistical tests was 5 % (*p* < 0.05).

## Results and discussion

Out of 128 patients evaluated, 109 met the inclusion criteria. Histology of CDT was papillary thyroid carcinoma in 91 patients (83.49 %), follicular thyroid carcinoma in 17 patients (15.59 %), and insular carcinoma in one patient (0.92 %). The mean follow-up time was 88 ± 70.6 months, ranging from 12 to 359 months since start of FT4 therapy. The baseline characteristics of the subjects in patients and control groups were comparable (including age, age at menopause, post-menopausal time, use of post-menopausal hormone therapy), except for mean TSH level (Table 1).

**Table 1:** Baseline characteristics of the patients with thyroid cancer and the control group

Variable	Patients	Control group	*P* value
Age (years-old)	58.43 ± 8.37	58.43 ± 8.40	0.968
Age at menopause (years-old)	47.19 ± 5.12	46.51 ± 5.33	0.235
Post-menopause time (years)	11.24 ± 9.54	11.93 ± 8.92	0.407
Caucasian (%)	92.6	91.74	0.801
Post-menopausal hormonal therapy (%)	19.2	27.5	0.150
BMI (kg/m^2^)	27.90 ± 4.51	28.33 ± 4.77	0.667
TSH (μIU/ml)	0.21 ± 0.28	2.27 ± 1.02	<0.001

The average calcium intake reported by the patients was 470.18 ± 346.69 mg/day, and the mean PTH value (n = 80) was 38.63 ± 13.02 pg/ml. Fifty-four patients (49.54 %) reported use of oral contraceptives in the past, for an average of 5.87 ± 4.85 years, and 19.20 % of the patients reported previous or current use of postmenopausal hormonal replacement therapy. The average percentage of follow-up time in which patients remained with more suppressed TSH levels (<0.001 μIU/ml) was 14 ± 12 %. During 39 ± 22 % of the follow-up time, the patients presented mean TSH level <0.1 μIU/ml. At the time of BMD evaluation, 24 patients (22.02 %) were being followed for 12–35 months, 20 (18.35 %) for 36–59 months, 39 (35.78 %) for 60–119 months, and 26 (23.85 %) for 120 months or more.

The mean T-scores of the patients were −1.09 ± 1.43 standard-deviations (SD) in L1 - L4 and −0.12 ± 1.18 SD in total femur. 19.2 % of the patients were diagnosed with osteoporosis, 40.4 % with osteopenia, and 40.4 % presented a normal BMD. The mean T-scores of the control group were −1.11 ± 1.3 SD in L1 - L4 and −0.37 ± 1.06 SD in total femur. The BMD diagnosis in the control group showed osteoporosis in 15.5 % of the women; osteopenia in 45 %, and 39.5 % presented a normal BMD. No significant differences were found between the lumbar (*p* = 0.940) or femoral (*p* = 0.105) T-scores of the patients and the control group. No significant differences were found between the two groups regarding the frequency of diagnosis of osteoporosis, osteopenia, or normal BMD (*χ*^2^ = 0.70, degree of liberty =2, *p* = 0.704).

**Fig. 1:**
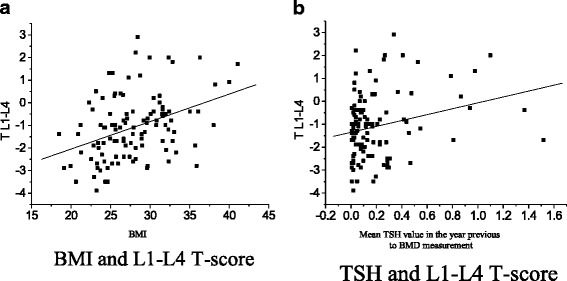
Factors with significant correlation to L1-L4 T-score in thyroid cancer patients under TSH supressive therapy. **a** BMI and L1-L4 T-score. **b** TSH and L1-L4 T-score

**Fig. 2:**
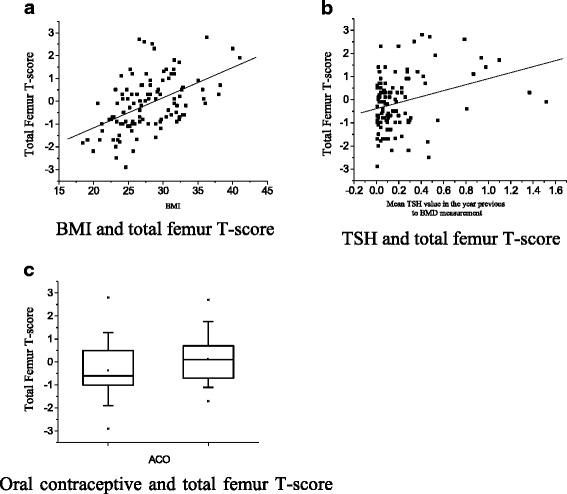
Factors with significant correlation to total femur T-score in thyroid cancer patients under TSH supressive therapy. **a** BMI and total femur T-score. **b** TSH and total femur T-score. **c** Oral contraceptive and total femur T-score

Univariate logistic regression identified significant positive correlations between T-score in L1-L4 and body mass index (BMI) (*p* < 0.001, R^2^ 0.1372) and between T-score in L1-L4 and the mean TSH values (*p* = 0.042 and R^2^ 0.038, Fig. 1a and b). Considering the T-score in total femur, there were significant correlations with BMI (*p* < 0.001, R^2^ = 0.2599), use of oral contraceptive in the past (*p* = 0.020, R^2^ = 0.0496) and mean TSH values (*p* = 0.010, R^2^ = 0.0605), as demonstrated in Fig. 2a, b and c.

Multivariate logistic regression analysis evidenced as factors significantly related to lower lumbar BMD: low BMI (*p* < 0.001; R^2^ = 0.15) and low mean TSH value (*p* = 0.021; R^2^ = 0.0423). For total femur, the factors that significantly affected the BMD were low BMI (*p* < 0.001; R^2^ = 0.26), low TSH value (*p* < 0.001; R^2^ = 0.07), and the history of never having used oral contraceptive (*p* = 0.007; R^2^ = 0.04). No relationship was found between BMD and mean serum FT4 levels. Furthermore, the total period of time in which patients remained with TSH levels <0.001μIU/ml or <0.1μIU/ml during follow-up had no significant association to BMD parameters. Only the TSH level measured in the year prior to BMD assessment showed correlation to BMD. Similar analyses in the control group evidenced significant positive correlation between BMI and L1-L4 T-score (*p* = 0.040; R^2^ = 0.0358) and between BMI and total femur T-score (*p* < 0.001; R^2^ = 0.1578).

Lumbar and spinal T-scores of postmenopausal women treated with exogenous LT4 therapy due to DTC were not lower than the T-scores of the control group with the same age and similar baseline characteristics in euthyroid state. This finding is in accordance with other previously published analyses, generally performed in smaller samples. Our analysis was based on a sample size calculation and included a greater number of postmenopausal women than most of the published studies.

A meta-analysis, published in 2006, assessed the risk of osteoporosis in DTC patients treated with TSH-suppressive doses of LT4, and included 14 cross-sectional studies (n = 10 to 44) and four longitudinal studies (n = 10 to 46) involving postmenopausal women [15]. The association between TSH-suppressive therapy and reduced BMD was found in only four out of 14 cross-sectional studies and two out of four longitudinal studies [4, 16, 17]. Eftkhari et al. also reported no significant association between TSH suppressive therapy and BMD reduction in 33 postmenopausal women compared to an age-matched euthyroid control group [8]. Lee et al. analyzed the BMD of a group of 94 Korean women receiving LT4 as part of treatment of DTC (66.7 % of them in postmenopausal state). They were divided into three groups according to TSH levels (≤0.001 μIU/mL, between 0.001 and 0.17 μIU/mL, and >0.17 μIU/mL) and no significant decrease was detected in BMD or bone turnover markers according to TSH level or even FT4 level. Also, the prevalence of osteoporosis and osteopenia was not different among groups [11].

A prospective study compared 144 women with TSH-suppressive therapy for DTC (mean TSH level 0.07 ± 0.10 μIU/ml) with 127 women with DTC without TSH-suppression treatment (mean TSH level 3.14 ± 1.69 μIU/ml) and demonstrated that BMD were lower only in patients ≥50 years-old that were treated with TSH-suppressive therapy for at least one year [6]. More recently, investigators from the Memorial Sloan-Kettering Hospital, in the United States, demonstrated that in a group of 771 patients (569 women, mean age 48 ± 14 years-old) with CDT at ATA low or intermediate risk of recurrence, followed by 6.5 years, the patients treated to reach a median TSH level ≤0.4 mIU/L were at increased risk of a composite outcome of atrial fibrillation and osteoporosis compared to those not suppressed [7]. Although the postmenopausal state of the studied women cohort was not described, the authors reported a synergistic effect between increasing age and TSH suppression [7].

Many reasons may explain the discrepancy between the results, such as different follow-up time, daily calcium intake or sunlight exposure, as well as characteristics of the different control groups. But one of the most important factors may be the different TSH levels observed in all those studies.

TSH receptors have been identified in osteoclasts and osteoblasts [18], and the role of TSH on bone metabolism has been studied. Abe et al. demonstrated that TSH inhibits osteoclastogenesis and also stimulates apoptosis of mature osteoclasts [19]. Besides that, they demonstrated that TSH can also inhibit osteoblasts’ differentiation, suggesting that TSH can modulate bone formation and resorption independently [19]. The authors showed that a 50 % reduction in the number of TSH receptors in mice could lead to osteoporosis. Although the animals were treated with thyroid hormones in the experiment, they exhibited a high rate of bone remodeling and decreased BMD, likely due to the deficient action of TSH on osteoblasts and osteoclasts [19]. Other animal studies confirmed that the absence of TSH signaling contributes to bone loss. Baliram et al. analyzed the skeletal phenotypes of wild-type and TSH-receptor knockout mice that were rendered hyperthyroid. They found that the hyperthyroid mice without TSH receptor had greater bone loss and resorption than hyperthyroid wild-type mice and they also identified a TSH-like factor that may confer osteoprotection [20]. Sun et al. further evaluated the mechanisms by which deficient TSH signaling affects the bone and reported that the skeletal actions of TSH deficiency are mediated, in part, through TNFα, in mice [21].

The hypothesis of a direct association of TSH levels and BMD was validated in a large cohort of postmenopausal women in the third “U.S. National Health and Nutrition Examination Survey” (NHANES III). The study showed a significant association between higher TSH levels and higher BMD, even when TSH levels were within the normal range [22]. Baqi et al. also showed that in a group of 114 postmenopausal women, those with normal TSH level had higher BMD than those with TSH level below the normal range, regardless of the FT4 level [23]. These findings are consistent with the hypothesis that TSH may have a direct effect on BMD in postmenopausal women. Similarly, data from the present study showed a direct association between TSH levels and BMD.

In our study, mean TSH levels assessed in the year prior to BMD analysis was not so suppressed because we included patients who were being followed by long times (mean follow-up time of 7.3 years). Such patients would not require very intensive TSH suppression, according to the guidelines [1]. Nonetheless, all of our patients have gone through a period of more suppressed TSH level after initial treatment and we could quantify the amount of time they were exposed to very low and moderately low TSH level. The number of months the patients were exposed to different degrees of TSH suppression was not correlated to patients’ BMD, suggesting the potential dynamic characteristic of bone tissue. It is possible to argue that low TSH level can reduce BMD in the short-term and there could be a possible recovery in BMD after increasing TSH levels during their follow-up. This would explain why only the TSH measured in the year before BMD assessment was related to lower BMD, and not the number of months that the patients were exposed to lower suppressed TSH level.

It was already shown the possibility of recovering the lumbar and femoral BMD in patients with reverted hyperthyroidism after one year of carbimazole treatment, including postmenopausal women [24], as well as the partial recovery of BMD after 7.5 months of treatment with methimazole [25]. Also in postmenopausal women with subclinical hyperthyroidism who were treated with radioiodine therapy, a prospective study showed an increase in lumbar and femoral BMD one year after TSH level were normal, and the recovery remained for up to two years of follow-up [26]. Another prospective study performed with 16 postmenopausal women with subclinical hyperthyroidism showed an increase in the forearm BMD two years after the normalization of TSH level with methimazole treatment [27]. Similarly, the postmenopausal patients with exogenous subclinical thyrotoxicosis evaluated in our study could also have recovered BMD after some increase in TSH level during follow-up. The BMD assessment in the moment when TSH was less suppressed (average of 0.21 ± 0.28 μUI/ml in this study) was associated to higher average T-scores. Possibly, if evaluation was performed in a moment when average TSH was lower, the average T-scores could have been lower.

In an attempt to identify other predictive risk factors for low BMD in our patients, we also examined characteristics that could be related to bone metabolism. Low BMI, which is a known risk factor for low BMD [12, 14], was associated with lower lumbar and spinal BMD, both independently and in association with lower TSH level. Interestingly, the mean BMI of the patients and the control group was in the overweight range, which could be considered even a protective factor for BMD [14].

Limitations of our study include the lack of follow-up BMD assessments to allow understanding of T-scores evolution during the time, but this was a cross-sectional study, similarly to the majority of the published studies on the subject. Although some patients had previous BMD evaluation, they were not performed in standardized methods to allow comparisons. Another limitation was the heterogeneity of the patients, including follow-up from 1 to more than 10 years, and this can explain the less suppressed mean TSH levels. Inclusion of patients with one year of follow-up seems to be adequate, since Sugitani et al. demonstrated decreased BMD in postmenopausal women with at least one year of TSH-suppressive therapy [6]. Despite the low average daily calcium intake referred by the patients, this was not correlated with low BMD, and patients were oriented to adequate their calcium intake. Other analysis, such as serum vitamin D levels and serum FT3 levels were not performed in this study, and PTH measurements were performed only in patients, not in control group. So, the interpretation of such data was limited in our study.

## Conclusions

Although we could not identify differences in the BMD of postmenopausal women under LT4 therapy for DTC *versus* the age-matched control group, we identified that the main risk factors for low lumbar and femoral BMD in that population were BMI and TSH levels, as well as oral contraceptive previous use for femoral BMD. Considering the results of the current study, we suggest that TSH may exhibits its deleterious effect on BMD only when levels are really suppressed. When TSH levels are raised, the BMD may be recovered. Our data reinforces previous findings, indicating that TSH suppression is a risk factor for osteoporosis, and the interventions in the treatment of DTC should avoid harmful effects, when possible.

## Abbreviations

TSH: Thyroid stimulating hormone
DTC: Differentiated thyroid cancer
BMD: Bone mineral density
BMI: Body mass index
SD: Standard-deviation
LT4: Levothyroxine
WHO: World Health Organization
